# Electrospun Polylactic Acid-Based Fibers Loaded with Multifunctional Antibacterial Biobased Polymers

**DOI:** 10.1021/acsapm.2c00928

**Published:** 2022-08-26

**Authors:** A. Chiloeches, R. Cuervo-Rodríguez, Y. Gil-Romero, M. Fernández-García, C. Echeverría, A. Muñoz-Bonilla

**Affiliations:** † Instituto de Ciencia y Tecnología de Polímeros (ICTP-CSIC), C/Juan de la Cierva 3, 28006 Madrid, Spain; ‡ Escuela Internacional de Doctorado de la Universidad Nacional de Educación a Distancia (UNED), C/Bravo Murillo, 38, 28015 Madrid, Spain; § Facultad de Ciencias Químicas, Universidad Complutense de Madrid, Avenida Complutense s/n, Ciudad Universitaria, 28040 Madrid, Spain; ∥ Hospital Universitario de Móstoles, C/Dr. Luis Montes, s/n, Móstoles, 28935 Madrid, Spain; ⊥ Interdisciplinary Platform for Sustainable Plastics Towards a Circular Economy-Spanish National Research Council (SusPlast-CSIC), 28006 Madrid, Spain

**Keywords:** poly(lactic acid), polyitaconates, biobased polymers, antimicrobial fibers, triazolium, *N*-halamine, compostability

## Abstract

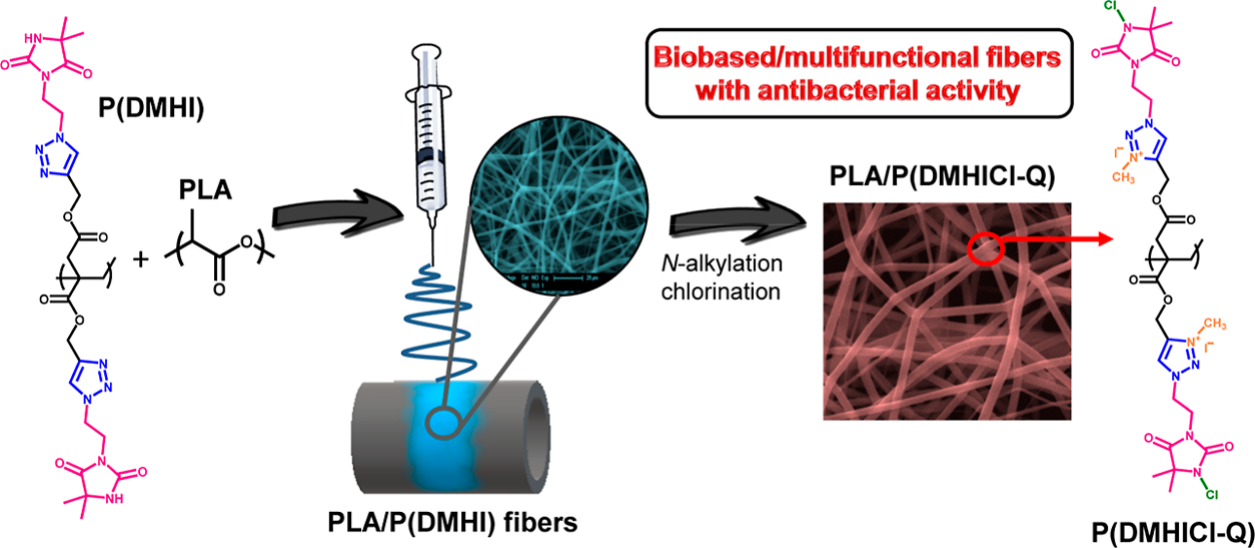

Here, we report the development of antibacterial and compostable electrospun polylactic acid (PLA) fibers by incorporation of a multifunctional biobased polymer in the process. The multifunctional polymer was synthesized from the bio-sourced itaconic acid building block by radical polymerization followed by click chemistry reaction with hydantoin groups. The resulting polymer possesses triazole and hydantoin groups available for further N-alkylation and chlorination reaction, which provide antibacterial activity. This polymer was added to the electrospinning PLA solution at 10 wt %, and fiber mats were successfully prepared. The obtained fibers were surface-modified through the accessible functional groups, leading to the corresponding cationic triazolium and *N*-halamine groups. The fibers with both antibacterial functionalities demonstrated high antibacterial activity against Gram-positive and Gram-negative bacteria. While the fibers with cationic surface groups are only effective against Gram-positive bacteria (*Staphylococcus epidermidis* and *Staphylococcus aureus*), upon chlorination, the activity against Gram-negative *Escherichia coli* and *Pseudomonas aeruginosa* is significantly improved. In addition, the compostability of the electrospun fibers was tested under industrial composting conditions, showing that the incorporation of the antibacterial polymer does not impede the disintegrability of the material. Overall, this study demonstrates the feasibility of this biobased multifunctional polymer as an antibacterial agent for biodegradable polymeric materials with potential application in medical uses.

## 1 Introduction

Microbial contamination on surface contacts of solid materials constitutes an important source of infections not only in biomedical devices, implants, and prostheses but also in other fields such as the food industry.^1,2^ Microbial adhesion onto such surfaces and subsequent biofilm formation, together with the emergence of antibiotic resistance, has become one of the more critical public health concerns.^3−5^ Then, surface disinfection and sterilization processes (physical and chemical) in hospitals, food processing facilities, or even in the domestic environment are crucial to reduce and control microbial infections. Typically, both physical and chemical methods are used. Application of chemical disinfectants, such as alcohols, chlorines, or quaternary amines, are effective treatments that, however, have several disadvantages, such as the need for high concentration to achieve sterility standards and the potential hazards to humans and the environment. For instance, common quaternary ammonium compounds such as the disinfectant benzalkonium chloride which generates bacterial resistance,^6^ have been found in sediments and soils.^7^ Similarly, the ethylene oxide chemical sterilization method presents potential hazards, as ethylene oxide is toxic, carcinogenic, and allergenic and can be present after the process.^8^ On the other hand, physical methods such as gamma irradiation, UV-irradiation, and steam sterilization can alter the properties of the materials, in particular polymer materials commonly used for biomedical or packaging applications.^9−11^ This is even more critical in biodegradable biopolymers such as polylactic acid (PLA), which typically have hydrolytic and thermal sensitivity, and sterilization can alter the properties of the materials.^12,13^ Nowadays, PLA is widely used as a sustainable alternative to conventional plastics^14,15^ in many applications, including the manufacture of biomedical devices and food packing. Indeed, a large part of plastic waste comes from these sectors, and therefore, the use of biodegradable and renewable polymers in such applications represents a great alternative to protect the environment.^16,17^

New perspectives to prevent or reduce surface contamination and bacterial growth on these bioplastic materials, maintaining safety requirements and long-term activity, are still challenged. Incorporation of antimicrobial biodegradable polymers into bioplastics is considered a valuable alternative, as these materials retain high long-term antimicrobial activity, do not leakage easily, have low toxicity and low susceptibility for developing resistance, and are able to degrade after their useful lifetime.^18−22^ Recently, our group has developed antibacterial biobased polymers derived from itaconic acid bearing cationic azolium groups derived from vitamin thiamine (B1). These polymers demonstrate excellent antibacterial activity against Gram-positive bacteria, negligible toxicity to human cells, and compostability.^23,24^ In addition, these antibacterial cationic copolymers have been studied as active additives in biodegradable films based on poly(butylene adipate-*co*-terephthalate) for packaging applications.^24^ However, the activity of these systems against Gram-negative bacteria is limited. Herein and based on our previous itaconate derivatives, we synthesize a biodegradable antibacterial polymer with multifunction, including contact-killing and release-killing mechanisms, with the purpose of improving the antibacterial activity and being more effective against Gram-negative bacteria. The multifunctional itaconate polymer was designed to combine in its structure two functional groups, cationic azolium and *N*-halamine groups. While the cationic groups in the polymer kill bacteria by physically damaging the cell membrane, the activity of *N*-halamine groups is due to the release of oxidative halogen, which can react with the appropriate biological receptors, affecting cell metabolism.^25^ This antibacterial biobased polymer was subsequently employed to impart antibacterial character to PLA fiber mats obtained by electrospinning in order to achieve “fully biodegradable” material. Electrospun fibers, widely used for food packages and biomedical materials such as face masks or wound dressing,^26−28^ have a large surface-to-volume ratio so, in principle, low quantities of antimicrobial polymer would be needed to achieve satisfactory results.^29^

## 2 Experimental Part

### 2.1 Materials

Itaconic acid (IA, ≥ 99%), propargyl alcohol (≥99%), 4-(dimethylamino) pyridine (DMAP, ≥ 99%), *N*,*N*′-dicyclohexylcarbodiimide (DCC, 99%), hydroquinone (99%), 5,5-dimethylhydantoin (97%), 1-bromo-2-chloroethane (98%), potassium hydroxide (KOH, 90%), sodium azide (NaN_3_, ≥99%), sodium iodide (NaI, 99.5%) di-*tert*-butyl dicarbonate (Boc_2_O, 99%), copper(I) chloride (CuCl, ≥99.995%), *N*,*N*,*N*′,*N*″,*N*″-pentamethyldiethylenetriamine (PMDTA, 99%), iodomethane (MeI, 99.5%), trifluoroacetic acid (TFA, 99%), *tert*-butanol (≥99.5%), acetic acid (≥99.7%), neutral aluminum oxide, sodium bicarbonate (NaHCO_3_, ≥99.7%), magnesium sulfate anhydrous (MgSO_4_, ≥99.5%), fluorescein sodium salt, sodium thiosulfate (Na_2_S_2_O_3_, 99%), cetyltrimethylammonium chloride (CTAC, ≥98%), iodine standard solution, anhydrous tetrahydrofuran (THF, 99.9%), and anhydrous *N*,*N*-dimethylformamide (DMF, 99.8%) were purchased from Merck and used as received. The radical initiator 2,2′-azobisisobutyronitrile (AIBN, 98%) was purchased from Acros and was recrystallized twice from methanol. PLA (6202D) was provided by NatureWorks. All the organic solvents were of AR grade; dichloromethane (DCM), THF, DMF, ethanol (EtOH), isopropyl alcohol (iPrOH), hexane, diethyl ether, and chloroform (CHCl_3_) were purchased from Scharlau; ethyl acetate (EtOAc) was purchased from Cor Química S.L.; toluene was purchased from Merck; sulfuric acid (H_2_SO_4_) was purchased from Panreac. Deuterated chloroform (CDCl_3_), water (D_2_O), and dimethyl sulfoxide (DMSO-*d*_6_) were acquired from Sigma-Aldrich. Cellulose dialysis membranes (CelluSep T1) were purchased from Membrane Filtration Products, Inc.

For the antibacterial assay: phosphate buffered saline powder (pH 7.4) and sodium chloride solution (NaCl suitable for cell culture, BioXtra) were purchased from Sigma-Aldrich. BBL Mueller–Hinton broth used as a microbial growth medium in the determination of the minimum inhibitory concentration (MIC) was acquired from Becton, Dickinson and Company, and the 96 well microplates were purchased from BD Biosciences. Columbia agar (5% sheep blood) plates were obtained from Fisher Scientific. American Type Culture Collection (ATCC): *Pseudomonas aeruginosa* (*P. aeruginosa*, ATCC 27853), *Escherichia coli* (*E. coli*, ATCC 25922), *Staphylococcus epidermidis* (*S. epidermidis*, ATCC 12228), and *Staphylococcus aureus* (*S. aureus*, ATCC 29213) were used as bacterial strains and were purchased from Oxoid.

### 2.2 Synthesis of poly(di(prop-2-yn-1-yl)) Itaconate, P(PrI)

The clickable polymer P(PrI) was synthesized as previously described.^23^ First, the monomer di(prop-2-yn-1-yl) itaconate (PrI) bearing clickable alkyne groups was synthesized via condensation reaction of itaconic acid with propargyl alcohol. Subsequently, the monomer PrI was polymerized by conventional radical polymerization with 5 mol % of the AIBN initiator, at a total concentration of 2 M in anhydrous DMF, at 70 °C under a nitrogen atmosphere for 24 h. The polymer **P(PrI)** was isolated by precipitation in isopropanol and dried overnight under vacuum at room temperature to afford a white solid (71% yield, *M*_n_ = 6700 g/mol, *M*_w_/*M*_n_ = 1.58). ^1^H NMR (400 MHz, CDCl_3_, δ, ppm): 4.67 (4H, −C*H*_2_C≡CH), 2.49 (2H, −CH_2_C≡C*H*), 1.99–1.00 (8H, CH_2_–CO and −CH_2_-chain).

### 2.3 Synthesis of *tert*-Butyl 3-(2-azidoethyl)-5,5-dimethylhydantoin-1-carboxylate, Boc-DMH-N_3_

First, 3-(2-azidoethyl)-5,5-dimethylhydantoin was synthesized through nucleophilic substitution reaction between 5,5-dimethylhydantoin and 1-bromo-2-chloroethane,^30^ followed by transformation of chlorine groups to azide groups via a substitution reaction. Briefly, 5,5-dimethylhydantoin (20.0 g, 156 mmol) and potassium hydroxide (8.7 g, 156 mmol) were dissolved in 100 mL of ethanol in a round-bottomed flask. Then, 1-bromo-2-chloroethane (26 mL, 312 mmol) was added, and the mixture was heated under reflux for 6 h. The reaction was cooled to room temperature, and the solvent was removed by rotary evaporation. The resulting white solid was dissolved in EtOAc and washed with water and NaHCO_3_ solution (10%) by several extraction steps. The organic fractions were dried over anhydrous magnesium sulfate, filtered, and evaporated under reduced pressure. The white solid was further purified by recrystallization in isopropanol/toluene (9:1) to afford **DMH-Cl** (yield 48%). ^1^H NMR (400 MHz, CDCl_3_, δ, ppm): 6.31 (s, 1H, −N*H*−), 3.85 (t, 2H, −C*H*_2_–Cl), 3.74 (t, 2H, −C*H*_2_–CH_2_–Cl), 1.46 (s, 6H, −C*H*_3_).

Later, chlorine-substituted hydantoin (DMH-Cl) (5.0 g, 26 mmol) was dissolved in DMF and deoxygenated by purging with argon. Sodium iodide (3.9 g, 26 mmol) and an excess of sodium azide (2.4 g, 37 mmol) were dissolved in 6 mL of water. Then, both solutions were mixed and heated at 80 °C under an argon atmosphere for 3 h. The resultant mixture was concentrated under reduced pressure and, then, was extracted with EtOAc and washed repeatedly with NaCl aqueous solution. The organic extract was separated, dried over MgSO_4_, and filtered, and the solvent was removed under reduced pressure to afford the product **DMH-N**_**3**_ as a colorless oil (yield: 88%). ^1^H NMR (400 MHz, CDCl_3_, δ, ppm): 6.57 (s, 1H, −N*H*−), 3.70 (t, 2H, −C*H*_2_–N_3_), 3.52 (t, 2H, C*H*_2_–CH_2_–N_3_), 1.44 (s, 6H, −C*H*_3_).

Subsequently, the amine group of the DMH-N_3_ product was protected by di-*tert*-butyl dicarbonate. DMH-N_3_ (0.9 g, 4.6 mmol), Boc_2_O (1.3 g 6 mmol), DMAP (56 mg, 0.46 mmol), and 25 mL of DCM were added in a round bottom flask. Then, the solution was stirred at room temperature for 4 h. The reaction mixture was washed with water several times. The organic layer was dried over Mg_2_SO_4_ and filtered, and the solvent was evaporated by rotary evaporation. The residue was purified by silica gel column chromatography using diethyl ether as eluent to give the protected product **Boc-DMH-N**_**3**_ (yield: 95%). ^1^H NMR (400 MHz, CDCl_3_, δ, ppm): 3.75 (t, 2H, −C*H*_2_–N_3_), 3.56 (t, 2H, C*H*_2_–CH_2_–N_3_), 1.64 (s, 6H, −C*H*_3_), 1.57 (s, 9H, −C*H*_3_ Boc). ^13^C NMR (100 MHz, CDCl_3_, δ, ppm): 174.9 (N–*C*=O), 151.8 (N–(*C*=O)–N), 148.4 (N–(*C*=O)–O), 84.4 (*C*_quat_, Boc), 60.3 (*C*_quat_, hydantoin), 48.3 (−*C*H_2_–N_3_), 38.2 (*C*H_2_–CH_2_–N_3_), 28.2 (−*C*H_3_, Boc), 23.2 (−*C*H_3_, hydantoin). FTIR: 2097 cm^–1^ associated with the azide group.

### 2.4 Synthesis of Poly(bis((1-(2-(3-(*tert*-butoxycarbonyl)-4,4-dimethyl-2,5-dioxoimidazolidin-1-yl)ethyl)-1*H*-1,2,3-triazol-4-yl)methyl)itaconate), P(Boc-DMHI)

The incorporation of the hydantoin Boc-DMH-N_3_ into the clickable itaconate polymer P(PrI) was conducted by click chemistry Cu(I)-catalyzed azide–alkyne cycloaddition (CuAAC). In a typical procedure, a mixture of polymer P(PrI) (1.50 g, 7.3 mequiv of alkyne groups), Boc-DMH-N_3_ (5.40 g, 18.2 mmol), PMDTA (300 μL, 1.45 mmol), and CuCl (0.072 g, 0.72 mmol) were dissolved in 40 mL of CHCl_3_. The mixture was stirred at 40 °C for 24 h and then passed through a neutral alumina column. The resulting polymer **P(Boc-DMHI)** was isolated by precipitation in ethanol, and the degree of modification was almost quantitative (yield: 98%). ^1^H NMR (400 MHz, DMSO-*d*_6_, δ, ppm): 8.08 (2H, *H*-triazole), 5.04 (4H, O–C*H*_2_-triazole), 4.61 (4H, C*H*_2_–N triazole), 3.85 (4H, C*H*_2_–N hydantoin), 1.49 (s, 18H, −C*H*_3_ Boc), 1.45 (s, 12H, −C*H*_3_), 2.66–1.00 (8H, C*H*_2_–CO and −C*H*_2_-chain).

### 2.5 *N*-Alkylation of P(Boc-DMHI) and Deprotection Reaction. Synthesis of Cationic Polymer P(DMHI-Q)

The polymer P(Boc-DMHI) was modified by *N*-alkylation reaction with iodomethane (MeI) leading to the corresponding cationic itaconate polymer. The polymer (1.50 g, 3.75 mequiv of triazole groups) was dissolved in 25 mL of anhydrous DMF, and then, a large excess of MeI was added (1.2 mL, 18.75 mmol; ratio triazole groups/alkyl iodide ≈ 1:5). The mixture was deoxygenated with argon for 15 min, sealed, and then stirred at 70 °C for one week to achieve a high degree of modification. The resulting cationic polymer was purified by precipitation into *n*-hexane followed by dialysis against distilled water and finally was isolated by freeze-drying. The degree of quaternization was almost quantitative. Subsequently, the deprotection reaction was performed. For this purpose, the polymer was dissolved in trifluoroacetic acid (5 mL) and stirred at 50 °C for 2 h. After cooling down to room temperature, the cationic polymer **P(DMHI-Q)** was purified by dialysis against distilled water and then lyophilized to yield a white solid (96%). ^1^H NMR (400 MHz, DMSO-*d*_6_, δ, ppm): 9.10 (2H, *H*-triazole), 5.44 (4H, O–C*H*_2_-triazole), 4.78 (4H, C*H*_2_–N triazole), 4.37 (6H, N^+^C*H*_3_ triazole), 3.85 (4H, C*H*_2_–N hydantoin), 1.29 (s, 12H, −C*H*_3_), 2.66–1.00 (8H, C*H*_2_–CO and −C*H*_2_-chain).

### 2.6 Chlorination of P(DMHI-Q). Synthesis of Polymer P(DMHICI-Q)

Initially, *tert*-butyl hypochlorite was synthesized.^30^ Briefly, sodium hypochlorite solution (250 mL, 10 wt %) was placed in a round-bottomed flask and cooled in an ice bath. A solution of *tert*-butyl alcohol (10 mL) and glacial acetic acid (15.5 mL) was added under stirring. After 10 min, the organic layer of the mixture was extracted with a separatory funnel, and the aqueous layer was discarded. The organic layer was washed successively with an aqueous solution of NaHCO_3_ 10% and water, dried over CaCl_2_, and filtered (yield: 73%).

The chlorination of the P(DMHI-Q) polymer to afford the **P(DMHICl-Q)** multifunctional polymer was carried out using *tert*-butyl hypochlorite as follows: P(DMHI-Q) (1.50 g, 4.85 mequiv of hydantoin groups) and *tert*-butyl hypochlorite (2.25 mL, 19.4 mmol) were dissolved in 20 mL of H_2_O/*t*-butanol (1/4 v/v). Then, the mixture was stirred in dark for 24 h at room temperature and dried under a vacuum with a rotary evaporator to obtain the final *N*-halamine polymer **P(DMHICl-Q)** in a quantitative amount. The polymer was characterized by Fourier transform infrared (FTIR), and the oxidative chlorine (Cl^+^) content of the polymer was quantified through the iodometric/thiosulfate titration method.^31^ Briefly, 1.8 mg of P(DMHICl-Q) was dissolved in 1 mL of 1% aqueous potassium iodide solution for 10 min to form I_2_ (the color of the solution changed to yellow). Then, 100 μL of 1% starch solution was added to the polymer solution that changed to blue. This solution was titrated with 0.5 mN sodium thiosulfate solution (the color of the solution changed from blue to colorless). The Cl^+^ content of the polymer was calculated as follows
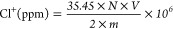
where Cl^+^ (ppm) is the weight percentage of the oxidative chlorine in the polymer, *N* (equiv/L) is the normality, *V* (L) is the volume of the titrant sodium thiosulfate, and *m* (g) is the weight of the polymer.

### 2.7 Electrospinning Process

Electrospinning solutions were prepared by dissolving the polymeric blends [PLA/P(DMHI) or P(DMHICl-Q)] in a 90/10 v/v mixture of CHCl_3_/DMF at a concentration of 18% w/v. Electrospun polymeric fibers were prepared from these solutions using a homemade electrospinner in a horizontal configuration equipped with a syringe needle connected to a high voltage power. The polymer solutions were fed at 1 mL h^–1^. The electrospun fiber mats were collected in a grounded aluminum foil collector located perpendicular at 12 cm from the needle tip by applying a voltage of 16 kV. Those conditions were selected from the results obtained in our previous works.^32,33^ The obtained electrospun samples, PLA/P(DMHI) and PLA/(DMHICl-Q), were dried for 48 h under a vacuum to remove any potential residual solvent before their use.

### 2.8 N-Alkylation and Chlorination of the Fibers. Preparation of Antimicrobial Fiber Mats

PLA/P(DMHI) fiber mats were subjected to *N*-alkylation and chlorination to obtain antimicrobial functional fibers containing triazolium and halamine groups at the surface. First, the PLA/P(DMHI) electrospun mat was cut into several pieces (1 × 1 cm^2^), and each of them was incubated in 1 mL of methanol. Then, a large excess of methyl iodide (200 μL) was added. The N-alkylation reaction was left for 10 days with constant shaking at 37 °C to ensure a complete reaction. After that period, the mats were washed with methanol several times to remove any residual reagent to afford PLA/P(DMHI-Q) fibers. Surface charge determination was performed following a method previously described in the literature.^34,35^ A Mat sample of 1 × 1 cm^2^ (2 cm^2^ surface area) was placed in 10 mL of 1 wt % aqueous sodium fluorescein solution for 10 min. After that, the sample was rinsed extensively with distilled water and sonicated to remove residual fluorescein. Then, the fluorescein was desorbed from the surface of the sample by treating the mat with 3 mL of 0.1 wt % CTAC solution for 20 min with shaking at 300 rpm. Subsequently, the amount of fluorescein obtained in the supernatant was determined by UV–vis spectroscopy (Lamda 35, PerkinElmer) in a solution prepared by adding 10% v/v of 100 mM phosphate buffer (pH 8.0). The absorbance of the resulting solution was measured at 501 nm, and the concentration of fluorescein was calculated with an extinction coefficient^36^ of 77 M^–1^ cm^–1^ assuming a relatioship of 1:1 for fluorescein to each accessible cationic triazolium group.

Next, the chlorination reaction of PLA/P(DMHI-Q) fibers was carried out to form *N*-halamine functional groups at the surface of the cationic fibers. For this reaction, the pieces of fibers obtained after alkylation were immersed in a diluted bleach solution (10% v/v) at room temperature for 2 h. Then, the samples were rinsed with a copious amount of water to reach pH 7 and dried to afford PLA/P(DMHICl-Q) fibers. The chlorine amount in the functionalized fibers was determined by the iodometric/thiosulfate titration method as described above^31^ using mat pieces of 1 × 1 cm^2^ (∼2.7 mg).

### 2.9 Characterization

NMR spectra were recorded on a Bruker Avance III HD-400AVIII spectrometer at room temperature using solvents CDCl_3_ and DMSO-*d*_6_. FTIR spectra were obtained with a PerkinElmer Spectrum Two instrument equipped with an attenuated total reflection module. Size exclusion chromatography measurements were performed on a Waters Division Millipore system equipped with a Waters 2414 refractive index detector. DMF stabilized with 0.1 M LiBr (Sigma-Aldrich, >99.9%) was used as eluent at a flow rate of 1 mL min^–1^ at 50 °C. The calibration was made with poly(methyl methacrylate) standards (Polymer Laboratories LTD). The morphology of electrospun fibers of PLA and PLA/itaconate polymers was studied using a scanning electron microscope (SEM) Philips XL30 with an acceleration voltage of 25–10 kV. The samples were coated with gold prior to scanning. SEM images were analyzed using NIH ImageJ software and measuring at least 100 fibers of each sample from different SEM images. Microhardness (MH) of the electrospun fibers was measured with a Vickers indentor attached to a Leitz microhardness tester. A contact load of 0.96 N and a time of 25 s were employed.

### 2.10 Antibacterial Assays

The antibacterial activity of the PLA/P(DMHICl-Q) fibers was measured following the E2149-13a standard method from the American Society for Testing and Materials (ASTM)^37^ against *P. aeruginosa*, *E. coli*, *S. epidermidis*, and *S. aureus*. First, bacterial cells were cultured on 5% sheep blood Columbia agar plates for 24 h at 37 °C. Then, the bacterial suspensions were prepared in saline using the McFarland turbidity scale and further diluted to 10^6^ colony-forming units (cfu) mL^–1^ with PBS. Next, PLA/P(DMHICl-Q) fiber mats (1 × 1 cm^2^) were placed in sterile falcon tubes containing 1 mL of the tested inoculum and 9 mL of PBS to reach a working solution of ∼10^5^ cfu mL^–1^. Control experiments were also performed in the presence of PLA fiber, and also in the absence of mats. The suspensions were shaken at 120 rpm for 30 min or 24 h. After this period, the colonies were counted by the plate counting method, and the reduction percentage was calculated in comparison to the control. The measurements were made at least in triplicate.

### 2.11 Disintegrability under Composting Conditions Test

The disintegrability test under simulated composting conditions of the prepared PLA and PLA/P(DMHICl-Q) fiber mats was performed at the laboratory scale level following the ISO 20200 standard.^38^ The synthetic wet compost was prepared by mixing 45 wt % of solid synthetic wet waste [10% of compost (Compo, Spain), 30% rabbit food, 10% starch, 5% sugar, 4% corn oil, 1% urea, and 40% sawdust] and 55 wt % of water contained in a perforated plastic box as composter reactors. Then, several pieces of mats from each sample (previously weighted) were placed in textile meshes buried in the compost and subjected to an aerobic disintegration process at 58 °C. Samples were withdrawn periodically, cleaned with distilled water, dried in an oven at 37 °C under a vacuum for 24 h, and reweighed. The disintegration degree was calculated by normalizing the sample weight, on different days of composting, to the initial weight. The samples were characterized by SEM and visual inspection.

## 3 Results and Discussion

### 3.1 Synthesis and Characterization of the P(DMHICl-Q) Antibacterial Polymer

**Figure 1:**
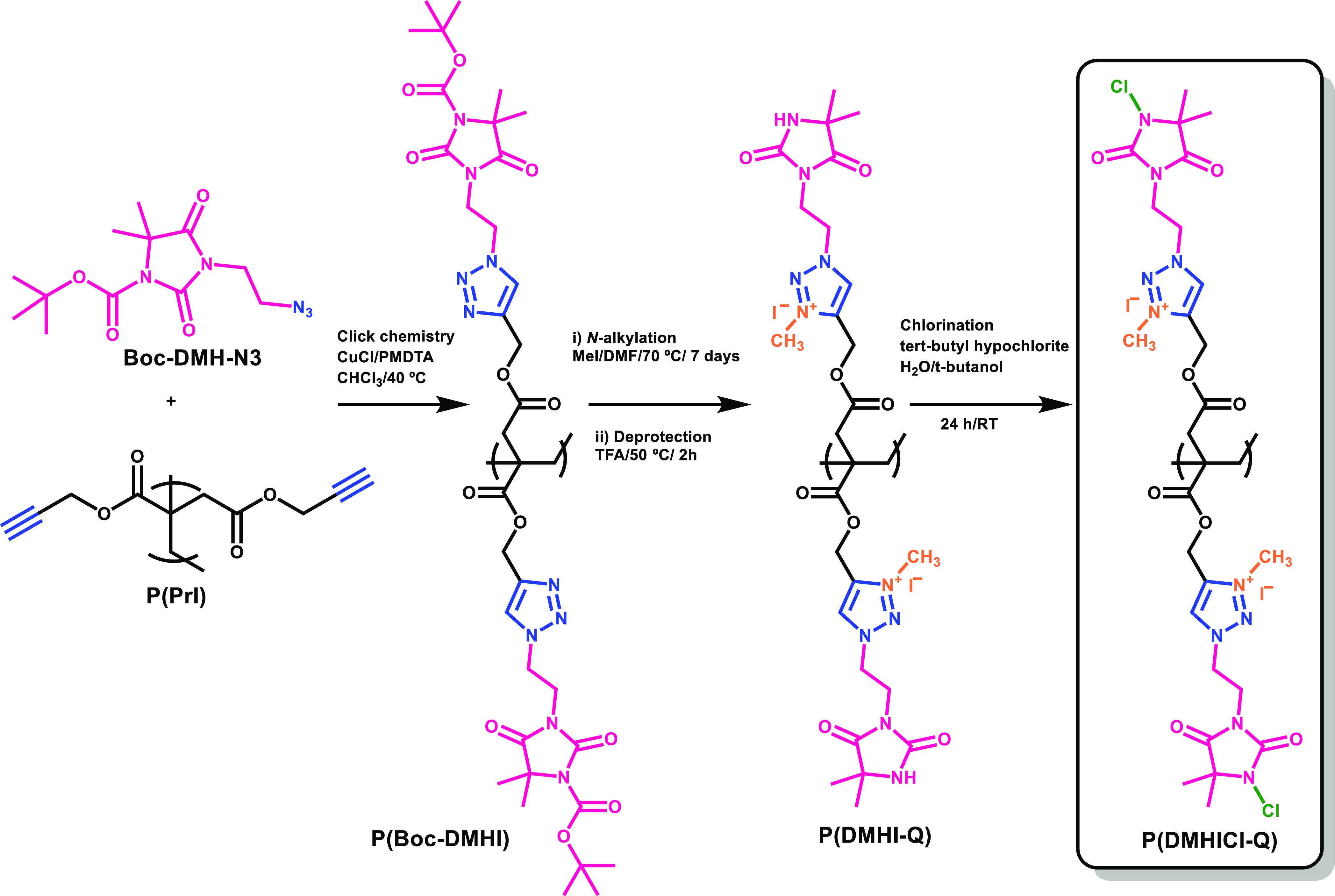
Synthesis route of the antibacterial biobased polymer P(DMHICl-Q).

In previous studies, biobased and biodegradable polymers derived from itaconic acid containing azolium antibacterial groups were prepared from a clickable polyitaconate developed by our group.^23^ These biopolymers bearing cationic thiazolium and triazolium groups demonstrated excellent antibacterial activity against Gram-positive bacteria and negligible toxicity to human cells but were poorly active against Gram-negative bacteria. Based on our previous finding, herein, we design and synthesize a multifunctional antibacterial polyitaconate bearing azolium and *N*-halamine groups to achieve systems with a broad antimicrobial spectrum. Figure 1 displays the synthetic route followed for the preparation of the antibacterial polymer P(DMHICl-Q). Clickable polymer derived from itaconic acid, P(PrI), was employed as a platform to prepare multifunctional polymers by azide–alkyne coupling reactions (CuAAC). For this purpose, Boc-protected 5,5-dimethylhydantoin functionalized with azide group (Boc-DMH-N_3_) was incorporated onto the polymer via CuAAC click chemistry, leading to triazole linkages. Subsequently, N-alkylating reaction of the triazole groups with methyl iodide provides the corresponding cationic itaconate derivatives. Each of these steps consisted of very efficient reactions and proceeded with high yields and a high degree of conversion of functional groups, as confirmed by FTIR and ^1^H NMR (see Supporting Information). After the N-Boc deprotection step, the chlorination reaction changes the amide group of hydantoin into *N*-halamines.

**Figure 2:**
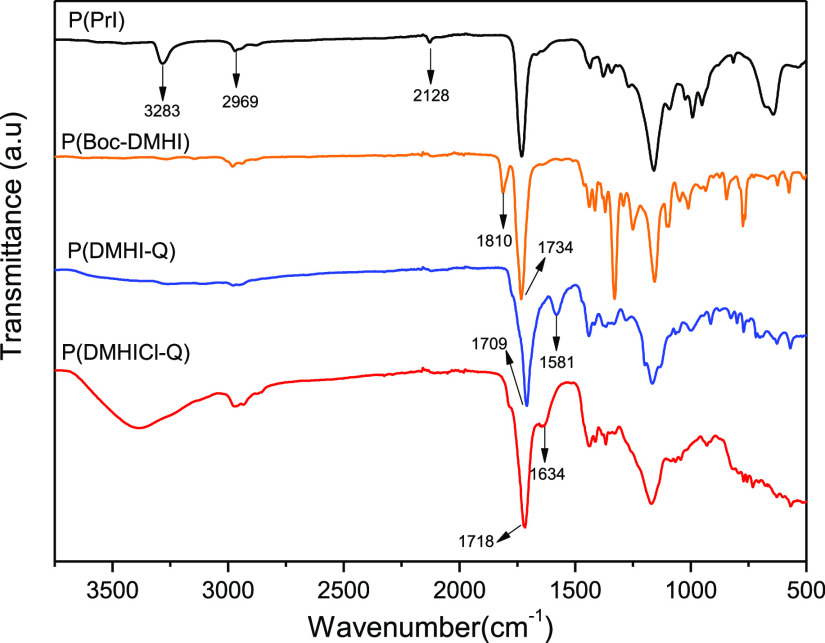
FTIR spectra of the antibacterial biobased polymer P(DMHICl-Q) and its precursors P(PrI), P(Boc-DMHI), and P(DMHI-Q).

Figure 2 shows the FTIR spectra of the P(DMHICl-Q) and their precursors. In the spectrum of P(PrI), the bands at 3283 and 2128 cm^–1^ assigned to the alkyne C–H and C≡C stretching vibrations, respectively, are clearly observed. After the click reaction of P(PrI) with the protected 3-(2-azidoethyl)-5,5-dimethylhydantoin (Boc-DMH-N_3_), new bands appear in the spectrum. It can be seen the band at 1810 cm^–1^ is associated with the ν (C=O) of the Boc group and the band at 1734 cm^–1^ due to the imide and ester C=O stretching vibrations. Subsequently, N-alkylation and deprotection reactions provide the cationic polymer P(DMHI-Q), in whose spectrum the bands assigned to the Boc group disappear and the vibrational band of the imide carbonyl bond is shifted to 1709 cm^–1^. Also, a new band at 1581 cm^–1^ corresponding to the ν(C=N^+^) clearly emerges, due to the formation of triazolium groups. The last chlorination step provides a shift of the imide carbonyl group to 1718 cm^–1^ as a result of the electron-withdrawing effect of the oxidative chlorine.^39^

It should be mentioned that synthetic *t*-butyl hypochlorite was used for chlorination instead of commercial bleach because, in this way, the polymer can be easily purified, as the excess of *t*-butyl hypochlorite is removed under vacuum.^40^ The content of oxidative chlorine in the polymer was iodometrically titrated and resulted in 7134 ppm for the tested polymer solution. This value corresponds to a degree of chlorination of 9%.

### 3.2 Preparation of Antimicrobial Electrospun Fibers based on PLA/P(DMHICl-Q)

**Figure 3:**
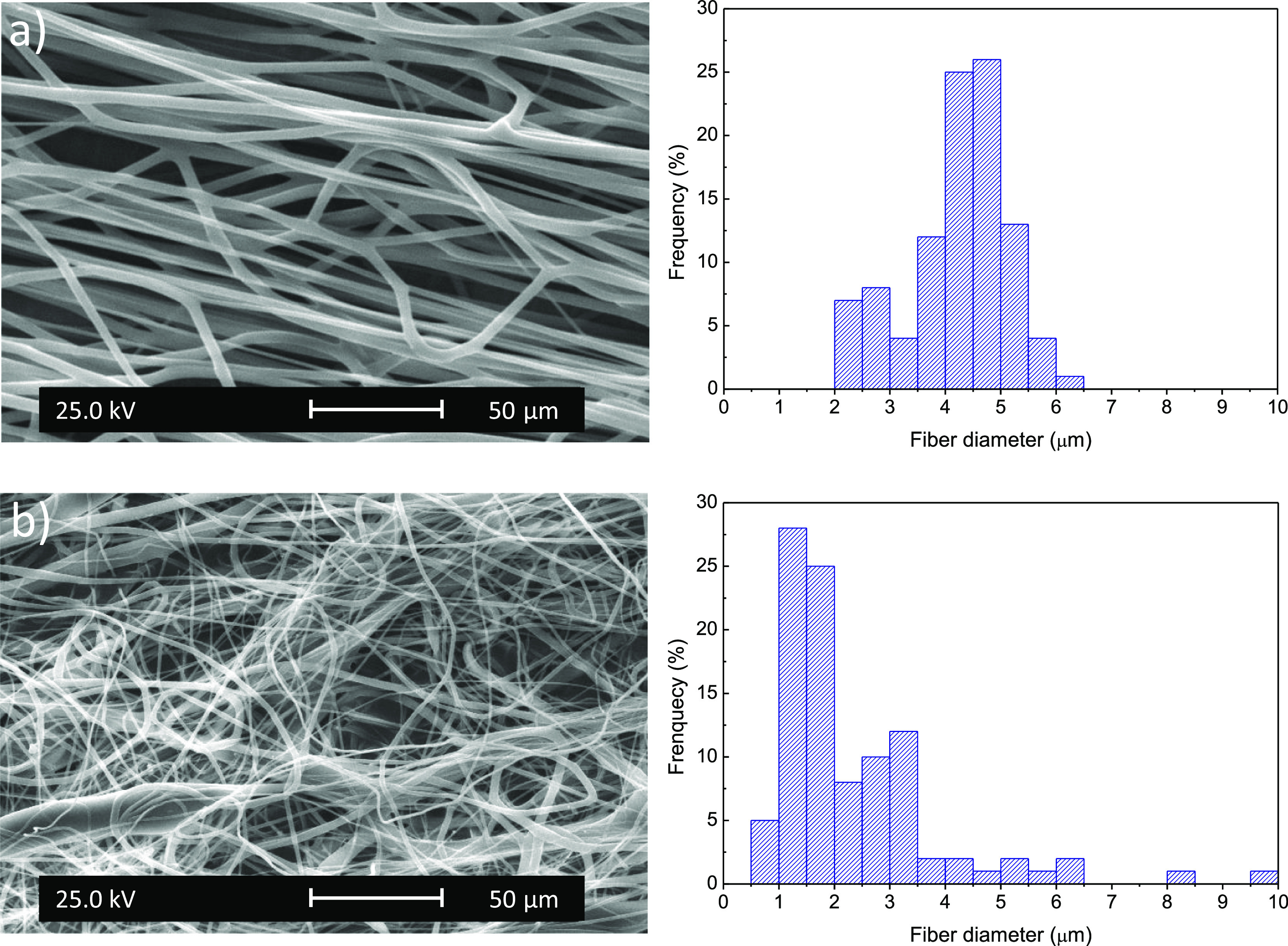
SEM micrographs and their corresponding histograms of fiber diameters of (a) PLA and (b) PLA/P(DMHI) fiber mats.

Next, the multifunctional biobased polymer P(DMHICl-Q) was employed to impart antibacterial activity to PLA electrospun fibers. First, the preparation of antimicrobial fibers by electrospinning technique was attempted from CHCl_3_/DMF solutions of PLA/P(DMHICl-Q) polymeric blends at a 90/10 ratio. However, the antibacterial polymer P(DMHICl-Q) containing cationic and halamine groups was not soluble in the solvent mixture, and its incorporation as a component of the fibers by the electrospinning process would conduct to maldistribution, aggregation, or leakage problems. It is well known that electrospinning of multiple components using one nozzle is critical and requires careful optimization of the systems. Indeed, this cationic polymer with halamine groups was not soluble in any solvent compatible with PLA or with the electrospinning process and therefore can hardly be electrospun in a direct way. For this reason, we follow an alternative approach consisting of the use of neutrally charged polymer resulting from the deprotection of P(Boc-DMHI). The P(DMHI) polymer was incorporated into the CHCl_3_/DMF solvent mixture at 10 wt % together with PLA 90 wt %, at a total polymer concentration of 18 wt %. Then, PLA-based fibers loaded with the polymer P(DMHI), bearing triazole and hydantoin groups, were prepared by electrospinning leading to antimicrobial precursor fibers. SEM images, shown in Figure 3, illustrate the morphology of the electrospun fiber mats compared to PLA fibers obtained under similar conditions. It is clearly observed that the incorporation of the P(DMHI) polymer affects the morphology of the fibers as it could modify the viscosity and conductivity of the solution, resulting in electrospun fibers with nonuniform size and shape. While the morphology of the electrospun PLA fibers was smooth, uniform, and bead-free, with a diameter of 4.2 ± 0.9 μm, the fiber mats loaded with P(DMHI) are nonuniform in size, with a lower average diameter of 2.3 ± 1.5 μm. Nevertheless, reasonable fiber mats can be obtained by this approach.

The fibers loaded with P(DMHI) polymer precursor were subsequently surface functionalized to provide antimicrobial activity. For this purpose, the N-alkylation reaction of the triazole groups was carried out with iodomethane to afford the corresponding cationic triazolium groups [P(DMHI-Q)], followed by chlorination of the hydantoin groups with bleach to provide *N*-halamine functionalities [P(DMHICl-Q)]. In this case, bleach was employed for the chlorination reaction because the washing steps of the fibers can be easily performed with water. The successful quaternization reaction on the surface was confirmed by measuring the accessible cationic units able to bind fluorescein. In this experiment, the fluorescein adsorbed on the surface, which corresponds to accessible positive surface charges, was desorbed with CTAC and quantified by UV–vis spectroscopy. The estimated cationic groups were found to be 1.4 × 10^15^ N^+^/cm^2^, a value near that previously determined for other contact-active surfaces.^34,41^ Likewise, the active chlorine content of the fiber mat (1 × 1 cm^2^, 2.7 mg), determined via the iodometric titration method, was calculated to be 1654 ppm, an amount enough, in principle, to impart activity to the surface, as has been demonstrated in other *N*-halamine-functionalized surfaces.^31^ These experiments demonstrated the successful functionalization of the fibers with both antimicrobial active groups.

### 3.3 Mechanical Properties

Microhardness measurements were carried out on the obtained PLA and PLA/P(DMHICl-Q) fiber mats to evaluate how the incorporation of the polyitaconate derivative and the posterior modification affects the mechanical behavior of PLA material. PLA fiber mats exhibit an MH value of 154 ± 5 MPa, near other values found in the literature for PLA materials;^42−44^ however, in PLA/P(DMHICl-Q) fibers, the MH increases to 171 ± 6 MPa. As expected, the addition of a glassy polymer such as this polyitaconate derivative results in a slight increase in the hardness of the materials, with values similar to PLA-based composites materials obtained by the incorporation of reinforcing fillers.^43,44^ It is also worth noting that the fiber diameter decreases with the incorporation of P(DMHICl-Q), which could also contribute to the differences in the mechanical properties.

### 3.4 Antibacterial Efficacy

Next, the antibacterial activity of the chlorinated and quaternized fiber mats, PLA/P(DMHICl-Q), was evaluated against Gram-positive, *S. epidermidis* and *S. aureus*, and Gram-negative bacteria, *P. aeruginosa* and *E. coli*, and compared with the quaternized fibers before chlorination, PLA/P(DMHI-Q), to analyze the combined action of both functionalities. In the antibacterial test, fiber mats of 1 × 1 cm^2^ were inoculated in 10 mL of bacterial suspension (10^6^ cfus). Controls of PLA fibers alone and inoculum without fibers were also tested. The control samples did not provide any noticeable reduction of bacteria after 24 h, whereas the fibers containing the antibacterial polyitaconate provoke great reduction (see Figure 2 of Supporting Information). Table 1 summarizes the percentage reduction of bacterial viable counts related to controls upon contact with antibacterial fibers after 30 min or 24 h. The results indicate that the fibers functionalized with cationic triazolium groups after 30 min of contact time are only effective against Gram-positive bacteria, causing 0.59 and 0.75-Log reductions of *S. epidermidis* and *S. aureus,* respectively. These films need a large contact time to achieve high bacterial reduction, 5-Log reductions, as described for other antibacterial systems based on cationic polymers acting through a contact kill mechanism, in which prolonged contacts are necessary to affect bacteria that are not in contact with the surface.^45^ This antibacterial efficacy is considerably reduced against *P. aeruginosa*, a Gram-negative bacteria, by 0.90-Log, whereas no effect on *E. coli* was observed after 24 h of contact time. Remarkably, the chlorination of the fibers considerably improves their activity for both Gram-positive and Gram-negative bacteria. Against Gram-positive bacteria, the chlorinated fibers yield 5-Log reduction after only 30 min of contact, whereas they cause 1.01-Log reduction of *P. aeruginosa* and 2-Log reduction of *E. coli*. The effectivity of these chlorinated PLA/P(DMHICl-Q) fibers practically reaches the highest results after the shortest time tested, 30 min.

**Table 1:** Antibacterial Activity Expressed as the Percentage of Bacterial Reduction (%) and Log Reduction (Log) of PLA/P(DMHI-Q) and PLA/P(DMHICl-Q) Against, *S. epidermidis*, *S. aureus*, *P. aeruginosa,* and *E. coli* After 30 min or 24 h of Contact Time

	*S. epidermidis*		*S. aureus*		*P. aeruginosa*		*E. coli*	
	%	Log	%	Log	%	Log	%	Log
PLA/P(DMHI-Q) (30 min)	75 ± 4	0.6 ± 0.1	82 ± 0.6	0.8 ± 0.1				
PLA/P(DMHI-Q) (24 h min)	99.999 ± 0.001	5.0 ± 0.3	99.999 ± 0.001	5.0 ± 0.1	88 ± 3	0.9 ± 0.1		
PLA/P(DMHICl-Q) (30 min)	99.999 ± 0.001	5.0 ± 0.2	99.999 ± 0.001	5.0 ± 0.1	90 ± 1	1.0 ± 0.2	99.0 ± 0.5	2.0 ± 0.2
PLA/P(DMHICl-Q) (24 h)	99.999 ± 0.001	5.0 ± 0.3	99.999 ± 0.001	5.0 ± 0.2	93.2 ± 0.6	1.2 ± 0.2	98.9 ± 0.6	2.0 ± 0.1

Thus, the incorporation of *N*-halamine with cationic triazolium groups demonstrates to be a good approach to achieving a broadened antibacterial spectrum of the fibers, improving the activity against both Gram-positive and Gram-negative bacteria.

### 3.5 Disintegrability

**Figure 4:**
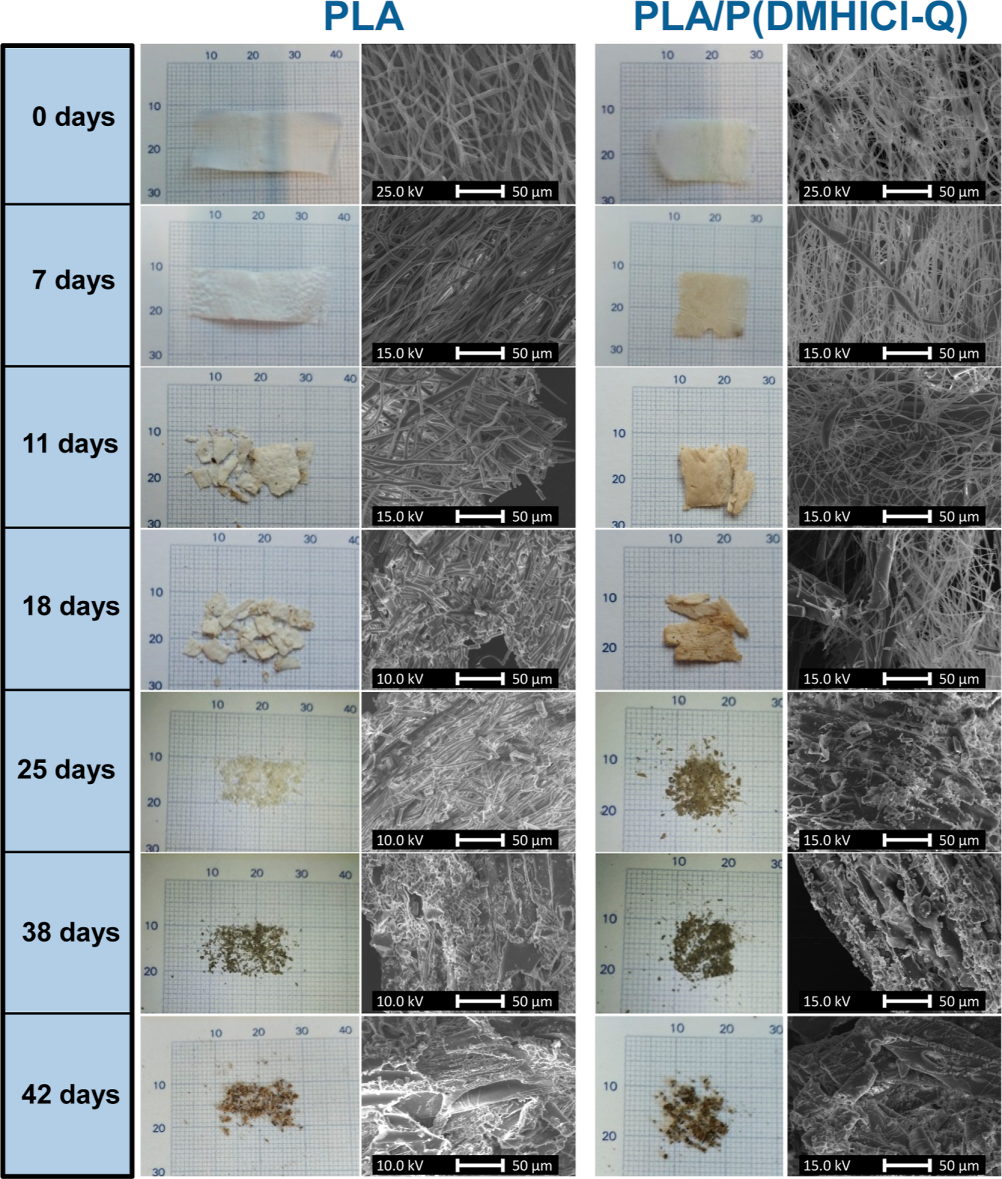
Visual appearance and SEM images of the tested PLA and PLA/P(DMHICl-Q) fiber mats over time under composting conditions.

To study the compostability of the antimicrobial polymeric fibers, a disintegration test was conducted under simulated industrial composting conditions at 58 °C according to the ISO 20200 standard.^38^ A qualitative evaluation of the physical disintegration of the fiber mats as a function of composting time was performed by taking photographs and by SEM measurements (Figure 4), confirming the biodisintegrable character of PLA and PLA-loaded mats in less than 90 days.

**Figure 5:**
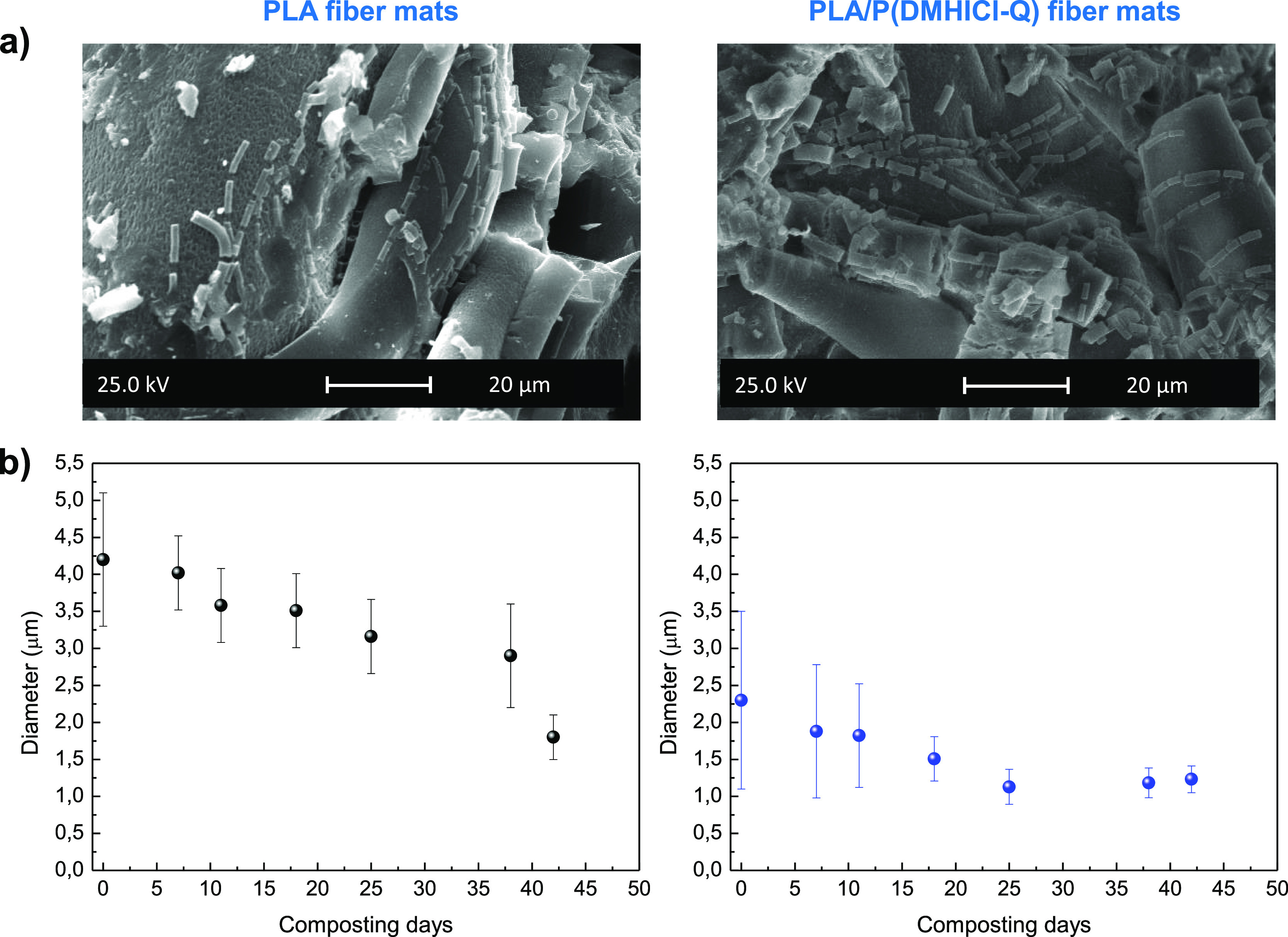
(a) SEM image of PLA and PLA/P(DMHICl-Q) fibers taken after 42 days of composting incubation. (b) Average fiber diameter of PLA and PLA/P(DMHICl-Q) fiber mats over time under composting conditions.

From the visual appearance of the samples, it is noticeable that the PLA/P(DMHICl-Q) sample turns yellowish on day 7, whereas the coloration becomes evident in PLA on day 11. This change in color may be due to hydrolytic degradation,^46,47^ in addition to the presence of sawdust. After 11 days under composting conditions, both samples exhibited considerable surface deformation and fractures, due to physical and/or chemical degradation of the polymers that cause a loss of flexibility. The PLA/P(DMHICl-Q) sample starts to turn dark brown, suggesting that the disintegration of this sample is faster than the degradation of the PLA mat. From day 25, only small pieces of mats were recovered, mostly smaller than 2 mm in size. At this point it is difficult to separate the polymeric sample from compost particles and, according to the ISO 20200 standard, pieces smaller than 2 mm should be discarded. Finally, after 42 days, samples reached a level of disintegration where no visible polymeric fragments could be easily recovered. Nevertheless, SEM images of the compost particles on day 42 reveal the presence, adhered on the surface, of small pieces of polymeric fibers in the range of micrometric scale (in diameter and length), demonstrating the existence of microplastic particles when apparently the fiber mats are completely disintegrated. Indeed, from SEM micrographs of the mats obtained at different composting times, a considerable fracture of fibers leading to a reduction of the length scale to micron can be appreciated. Figure 5a showed magnified SEM images of the PLA and PLA/P(DMHICl-Q) fibers taken at large incubation composting period, 42 days, in which is appreciated fiber with lengths smaller than 10 μm. Also, during the degradation process, a reduction in the fiber diameter is observed. Figure 5b displays the average fiber diameter as a function of composting time. In both samples, PLA and PLA/P(DMHICl-Q), the fiber diameter diminishes with the incubation periods, as previously observed in PLA fiber mats biodegraded under simulated physiological conditions.^48^ From SEM images of the samples obtained at different incubation times, as seen from visual observation, it seems that the disintegration process is a bit faster in PLA fibers loaded with the antimicrobial polymer P(DMHICl-Q), probably due to the smaller fiber diameter and, therefore, its larger surface area.

**Figure 6:**
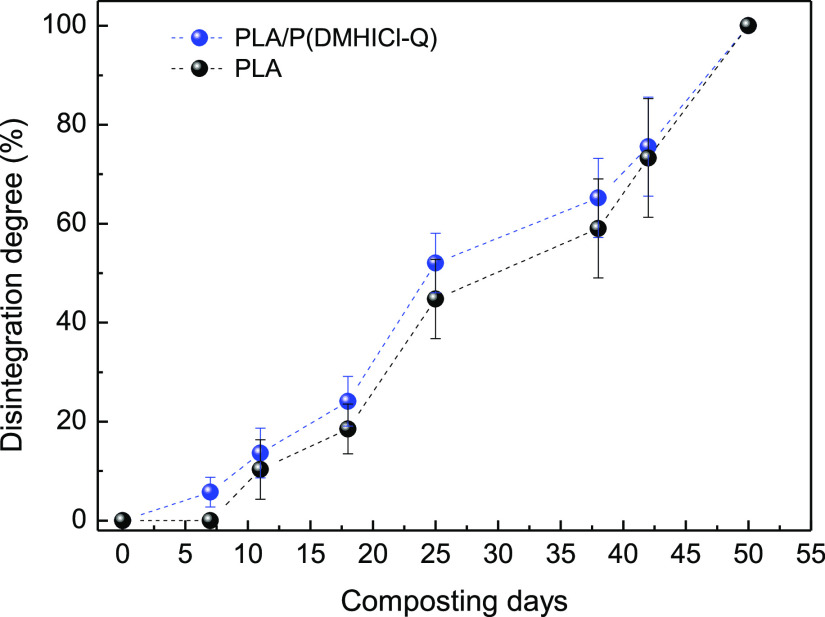
Disintegration degree of PLA and PLA/P(DMHICl-Q) fiber mats over time under composting conditions.

The disintegration degree was also analyzed in terms of mass loss as a function of disintegration time (Figure 6). The process seems to be slightly accelerated in the PLA/P(DMHICl-Q) samples, as previously observed in photographs and SEM micrographs. However, statistical analysis using one-way ANOVA followed by Tukey’s test (*p* < 0.05) indicates the degradation profile of PLA and PLA/P(DMHICl-Q) fibers are not significantly different.

## 4 Conclusions

In this study, antibacterial and compostable PLA-based fibers were successfully fabricated by incorporating a multifunctional biobased polymer derived from itaconic acid bearing *N*-halamine and triazolium antibacterial groups, P(DMHICl-Q). In the first attempt, the antibacterial polymer was synthesized by the attachment of both functionalities onto a polyitaconate derivative following efficient and straightforward approaches. However, the polymer exhibits poor solubility in common solvents used in the electrospinning process of PLA, which makes the preparation of homogeneous fibers very difficult. As an alternative, a precursor of such a polymer obtained before N-alkylation and chlorination, P(DMHI), was employed and added directly to the electrospinning PLA solution. Subsequent surface functionalization of the accessible triazole and hydantoin groups provides PLA-based fibers with the ability to efficiently inactivate Gram-positive and Gram-negative bacteria. It was demonstrated that the presence of cationic triazolium groups at the surface only provides high efficacy against Gram-positive bacteria, while their combination with *N*-halamine groups extends the effectiveness also to Gram-negative bacteria. Remarkably, due to the small diameter, and therefore high surface area, a minor amount of fibers loaded with antimicrobial is needed for pathogen inactivation. The compostability of the antibacterial electrospun fibers was also tested under simulated industrial composting conditions. It was observed that the incorporation of the antimicrobial biobased polymer apparently did not compromise PLA disintegration under aerobic composting conditions. However, a deep analysis of the compost samples proves the presence of microplastics in both samples (PLA and antimicrobial loaded PLA) after the biodisintegration test following the ISO 20200 standard protocol. Then, more studies are needed in this regard. In addition, these multifunctional biobased polymeric derivatives can be employed as an additive or component not only in electrospun fibers but could also be used in the preparation of films or other devices by different processing methods such as melt extrusion and additive manufacturing in which the solubility problem is avoided.

## Supporting Information Available

^1^H NMR spectra of the P(Boc-DMHI) and P(DMHI-Q) polyitaconate derivatives and photographs of the agar plates of *E. coli* (Gram-negative bacteria) and *S. epidermidis* (Gram-positive bacteria) bacterial colonies after spreading inoculum (at different dilutions) in the previous contact with fibers (PDF)
