# Supplementary material for: Electrospun Polylactic Acid-Based Fibers Loaded with Multifunctional Antibacterial Biobased Polymers
Source: ACS Appl Polym Mater. 2022 Aug 26;4(9):6543–52. doi: 10.1021/acsapm.2c00928 (PMC9799243; doi:10.1021/acsapm.2c00928)
Supplement: Supplementary file 1 — ap2c00928_si_001.pdf [file ap2c00928_si_001.pdf]

Supporting Information

Electrospun PLA based fibers loaded with multifunctional antibacterial biobased polymers

A. Chiloeches[1,2], R. Cuervo-Rodríguez[3], Y. Gil-Romero[4], M. Fernández-García[1,5], C. Echeverría[1,5]*, A. Muñoz-Bonilla[1,5]*

[1]Instituto de Ciencia y Tecnología de Polímeros (ICTP-CSIC), C/Juan de la Cierva 3, 28006 Madrid, Spain

[2]Escuela Internacional de Doctorado de la Universidad Nacional de Educación a Distancia (UNED), C/ Bravo Murillo, 38, 28015 Madrid, Spain

[3]Facultad de Ciencias Químicas, Universidad Complutense de Madrid, Avenida Complutense s/n, Ciudad Universitaria, 28040 Madrid, Spain

[4]Hospital Universitario de Móstoles C/ Dr. Luis Montes, s/n, 28935 Móstoles, Madrid, Spain.

[5]Interdisciplinary Platform for Sustainable Plastics towards a Circular Economy-Spanish National Research Council (SusPlast-CSIC), 28006, Madrid, Spain.

Email: cecheverria@ictp.csic.es (C. Echeverria), sbonilla@ictp.csic.es (A. Muñoz Bonilla).

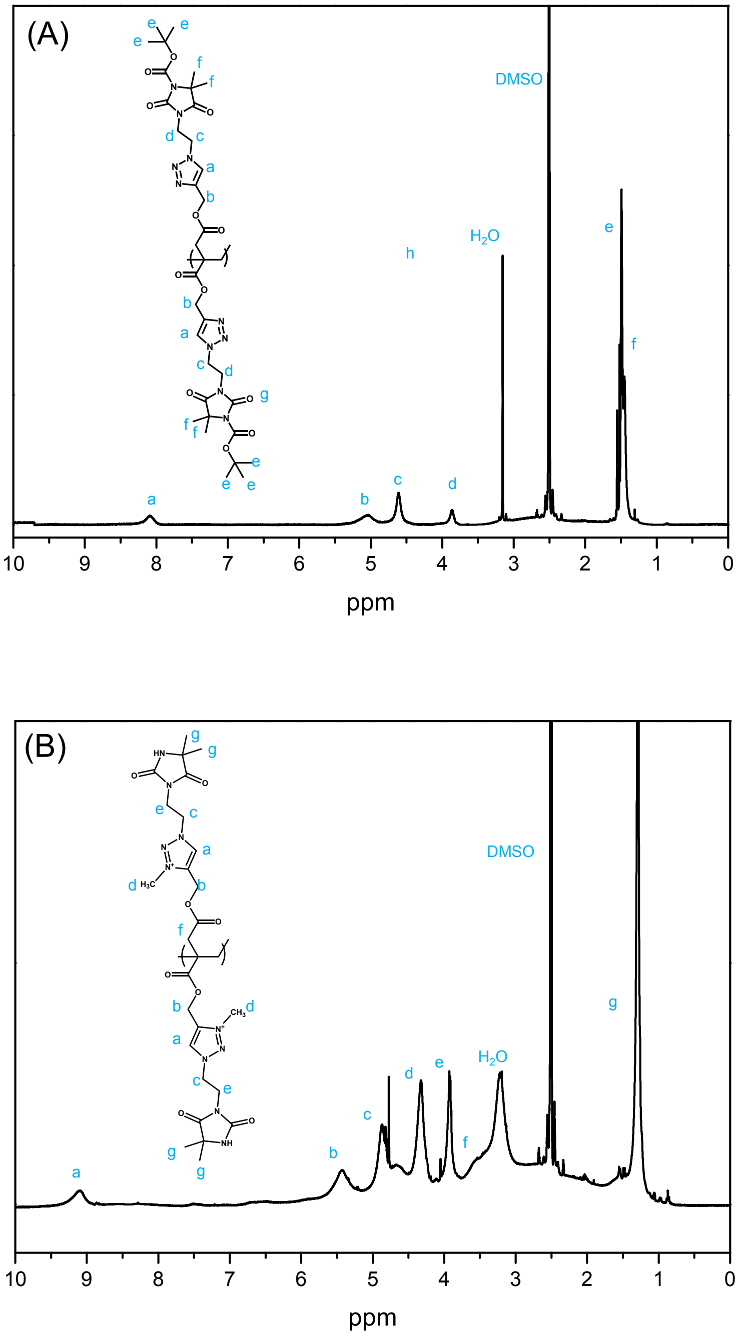

Figure S1. 1H-NMR spectra of (A) P(Boc-DMHI) and (B) P(DMHI-Q) polyitaconate derivatives obtained in DMSO-d_6 as solvent.

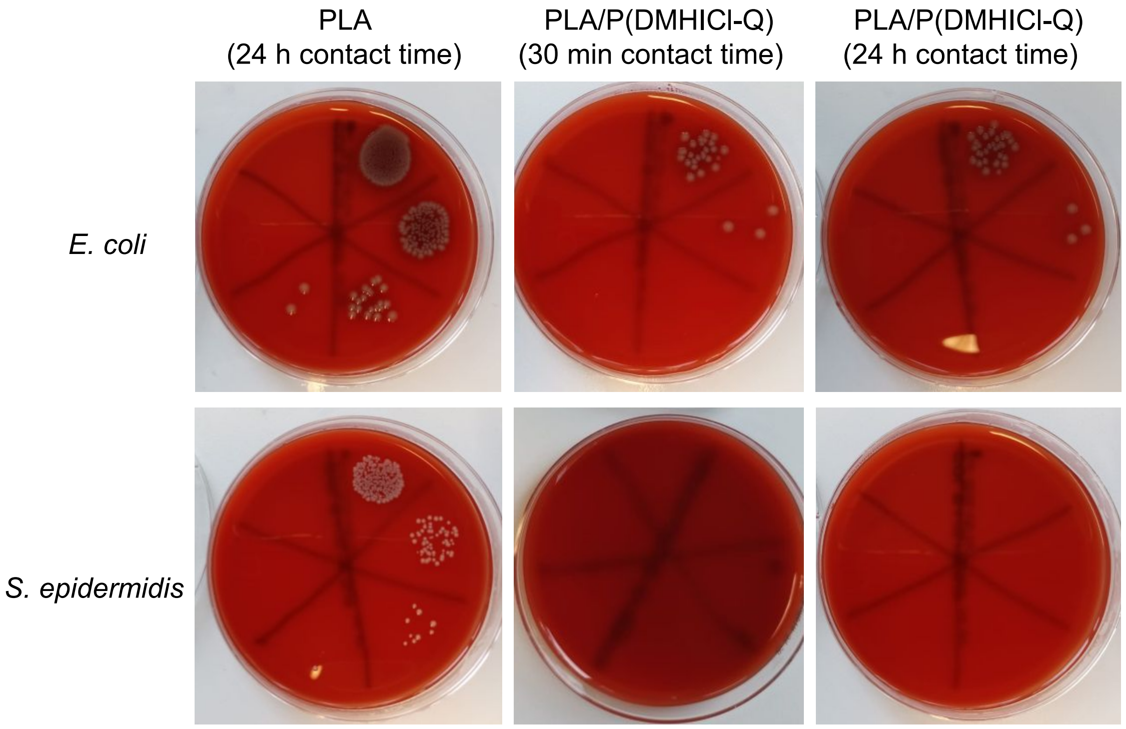

Figure S2. Pictures of the agar plates of *E. coli* (Gram-negative bacteria) and *S. epidermidis* (Gram-positive bacteria) bacterial colonies after spreading inoculum (at different dilutions) in previous contact with PLA fibers (control, left) and incubation during 24 h, and in contact with PLA/(P(DMHICl-Q) fibers and incubation during 30 min (middle) and 24 h (right).
